# Regulation of AUXIN RESPONSE FACTOR condensation and nucleo-cytoplasmic partitioning

**DOI:** 10.1038/s41467-022-31628-2

**Published:** 2022-07-11

**Authors:** Hongwei Jing, David A. Korasick, Ryan J. Emenecker, Nicholas Morffy, Edward G. Wilkinson, Samantha K. Powers, Lucia C. Strader

**Affiliations:** 1 Department of Biology, Duke University, Durham, NC 27008 USA; 2 Center for Engineering MechanoBiology, Washington University, St. Louis, MO 63130 USA; 3 Center for Science and Engineering Living Systems (CSELS), Washington University, St. Louis, MO 63130 USA; 4 Department of Biology, Washington University, St. Louis, MO 63130 USA

**Keywords:** Auxin, Proteasome

## Abstract

Auxin critically regulates plant growth and development. Auxin-driven transcriptional responses are mediated through the AUXIN RESPONSE FACTOR (ARF) family of transcription factors. ARF protein condensation attenuates ARF activity, resulting in dramatic shifts in the auxin transcriptional landscape. Here, we perform a forward genetics screen for ARF hypercondensation, identifying an F-box protein, which we named AUXIN RESPONSE FACTOR F-BOX1 (AFF1). Functional characterization of SCF^AFF1^ revealed that this E3 ubiquitin ligase directly interacts with ARF19 and ARF7 to regulate their accumulation, condensation, and nucleo-cytoplasmic partitioning. Mutants defective in *AFF1* display attenuated auxin responsiveness, and developmental defects, suggesting that SCF^AFF1^ -mediated regulation of ARF protein drives aspects of auxin response and plant development.

## Abstract (web summary)

Auxin-driven transcriptional responses are mediated by ARF transcription factors. Here the authors characterize an F-box protein, AFF1, that regulates the accumulation, condensation, and nucleo-cytoplasmic partitioning of ARF19 and ARF7.

## Introduction

The plant hormone auxin pivotally regulates plant growth. The AUXIN RESPONSE FACTOR (ARF) family of transcription factors mediate auxin responses to drive many developmental events as a function of auxin transcriptional output^1^. Protein condensation attenuates ARF activity to regulate auxin response competence in a developmentally-relevant context^2^. In Arabidopsis, certain Class-A ARFs, such as ARF7 and ARF19, undergo protein condensation. ARF7 and ARF19 are localized to nuclei of cells in which active growth is occurring and are diffusely localized within these nuclei. Conversely, these transcription factors are held in cytoplasmic condensates in cells that have ceased active growth, such as cells of the upper root. A single point mutation in the ARF19 PB1 domain was sufficient to disrupt protein condensation, resulting in constitutive ARF19 protein localization, expanded zones of auxin competence, and dramatically increased auxin transcriptional responses. This study suggested that ARF7 and ARF19 condensation acts as a major mechanism to regulate transcriptional activity.

Although ARF condensation regulates cellular competence for auxin responses, mechanisms controlling ARF condensate formation were unknown. To address these knowledge gap, we designed a forward genetics screen for mutants that display increased ARF19 condensation. We identified a mutant defective in a gene encoding an F-box protein, which we named *AUXIN RESPONSE FACTOR F-BOX1* (*AFF1*). AFF1 physically interacts with ARF19 and the closely-related ARF7. Further, *aff1* mutants, which exhibit increased numbers and sizes of cytoplasmic ARF7 and ARF19 condensates, accompanied by increased ARF7 and ARF19 accumulation, display attenuated auxin responsiveness and morphological abnormalities. Our results support a model in which SCF^AFF1^ promotes both ARF degradation and ARF nuclear localization to prevent inappropriate protein condensation and to maintain auxin responsiveness.

## Results

### Identifying AUXIN RESPONSE FACTOR F-BOX1 (AFF1)

**Fig. 1:**
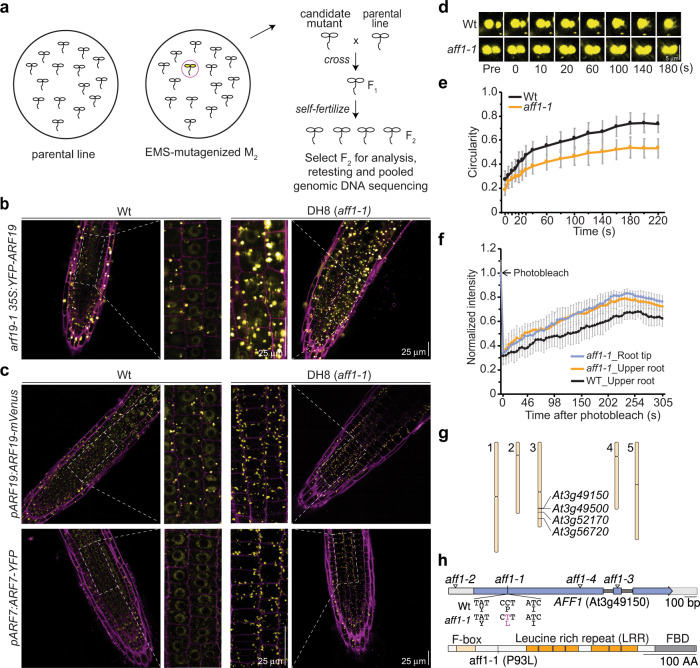
Identification *At3g49150*/*AFF1* for ARF19 hypercondensation. **a** EMS-mutagenized M_2_ seeds of *arf19-1 35* *S:YFP-ARF19* were screened for individuals with increased numbers of YFP-ARF19 condensates using a fluorescence dissecting microscope. Isolate DH8 was backcrossed to the parental line (*arf19-1 35* *S:YFP-ARF19*) and the resultant F_2_ individuals displaying YFP-ARF19 hypercondensation identified and used for whole-genome sequencing of bulk segregants. **b** Confocal images of 3d-old Wt (Col-0) and DH8 (*aff1-1*) seedlings carrying *35* *S:YFP-ARF19* (false-colored yellow) with cell walls counterstained with propidium iodide (false-colored magenta). Scale bar = 25 µm. **c** Confocal images of 3d-old wild type (Wt; Col-0) and *aff1-1* seedlings carrying *pARF19:ARF19-mVenus*, or *pARF7:ARF7-YFP* (false-colored yellow) with cell walls counterstained with propidium iodide (false-colored magenta). Scale bar = 25 µm. **d** Time course confocal images showing fusion of condensates in root transition zone cells of 3d-old Wt (Col-0) and *aff1-1* seedlings carrying *UBQ10:YFP-ARF19*. Scale bar = 5 µm. **e** Condensate circularity measurements after condensate fusion events in 3d-old Wt (Col-0) and *aff1-1* seedlings carrying *UBQ10:YFP-ARF19* (mean ± SD; *n* = 20). **f** Half-condensate FRAP recovery profiles after photobleaching condensates from 3d-old Wt (Col-0) and *aff1-1* seedlings in root tip or upper root region carrying *UBQ10:YFP-ARF19* (mean ± SD; *n* = 20). **g** Map positions of homozygous EMS-caused mutations identified in DH8 bulk segregants. **h** *At3g49150*/*AFF1* schematic depicting the exons (blue), UTRs (gray), and introns (black). Locations of the *aff1-1* point mutation and *aff1–2* (Salk_053818), *aff1–3* (Salk_083453), and *aff1–4* (Sail_427_G06) insertion sites are indicated. *AFF1* encodes a putative F-box protein with an N-terminal F-box domain, leucine rich repeat (LRR) region, and C-terminal F-box domain (FBD) motif. Three independent experiments were performed for (**b**), (**c**) and (**d**) with similar results. The source data for (**e**) and (**f**) are provided as a Source Data files.

ARF7 and ARF19 are class-A ARFs that act as transcriptional activators of auxin response and coordinately play essential roles in plant development^3^. Activity of these closely-related proteins is regulated by protein condensation^2^. To identify factors regulating their condensation, we carried out a forward genetics, fluorescence-based, screen of EMS-mutagenized *arf19-1 35* *S:YFP-ARF19* for individuals displaying increased ARF19 condensation (Fig. 1a). Unlike our previous report of YFP-ARF19 localization when driven behind the *UBQ10* promoter or of ARF19-mVenus localization when driven behind the *ARF19* native promoter^2^, overexpression of YFP-ARF19 behind the strong constitutive *35* *S* promoter resulted in multiple ARF19 condensates in the root tip (Fig. 1b). The heightened level of ARF accumulation provided by the strong *35* *S* promoter allowed for easy visualization of YFP-ARF through a dissecting microscope, enabling the screen for increased YFP-ARF19 condensation.

From this screen, isolate DH8 (*aff1-1*) displayed increased numbers and sizes of ARF19 condensation (Fig. 1b). In addition, the DH8 (*aff1-1*) mutation conferred hyperaccumulation of ARF19-mVenus and ARF7-YFP when driven behind their respective native promoters (Fig. 1c) and when driven behind the *UBQ10* constitutive promoter (see below and Supplementary Fig. 4).

To better understand the material properties of ARF19 condensates with wild type and *aff1*, we examined condensate fusion events. We previously showed that when YFP-ARF19 condensates fuse in young cells of wild type, they rapidly readopt spherical morphologies whereas fusion of YFP-ARF19 condensates in older cells of wild type results in amorphous multilobed assemblies that retain ultrastructure from pre-fusion condensates^2^. Because our previous work demonstrated low internal molecular dynamics of ARF19 within condensates from the oldest cells within the root^2^ and also that ARF19 condensates undergo a rapid aging process^4^, we examined condensate fusion events in the first root epidermal displaying a root hair bulge – this cell marks a transition from predominantly nuclear signal to the formation of condensates in wild type and allowed us to assess condensates in the same age of cell with a higher probability of being recently formed. We found that YFP-ARF19 condensates in this cell formed larger spherical bodies upon fusion in wild type (Fig. 1d, e). In contrast, YFP-ARF19 condensates in *aff1* retained distinct ultrastructure post-fusion and failed to achieve the same post-fusion sphericity as condensates in wild type (Fig. 1d, e), suggesting decreased dynamics within ARF19 condensates in *aff1*.

Condensate morphology is often linked to condensate dynamics. We therefore performed half-condensate fluorescence recovery after photobleaching (FRAP) experiments to assess the mobility of ARF19 within condensates of wild type and *aff1* carrying *UBQ10:YFP-ARF19*. In first root epidermal cell exhibiting a root hair bulge, we found that YFP-ARF19 within condensates from *aff1* displayed higher levels of recovery than that of YFP-ARF19 within condensates from wild type (Fig. 1f), suggesting that ARF19 mobility within these assemblies is greater in *aff1* than in wild type. We also performed half-condensate FRAP of ARF19 condensates in epidermal cells within the root meristem of *aff1* (a region in which there are rarely ARF19 condensates in wild type expressing *UBQ10:YFP-ARF19*), finding similar recovery after photobleaching in these cells as we did in the slightly older epidermal cells at the root hair bulge. Thus, even though ARF19 condensates within *aff1* are slow to form spherical bodies after fusion (Fig. 1e) and often retain the distinct ultrastructure of pre-fusion bodies (Fig. 1d), recovery after photobleaching is more rapid than in wild type, which may be a reflection of the higher overall level of YFP-ARF19 protein in the system and likely increased availability of YFP-ARF19 protein to exchange with the dilute phase.

We used a whole-genome sequencing of bulk segregants approach^5^ to identify the causative mutation in DH8, uncovering four homozygous, EMS-related mutations (Fig. 1g). Because protein condensation is a concentration-dependent process^4^, we hypothesized that the mutation in *At3g49150*, encoding a putative F-box protein, was likely causative. We named this gene *AUXIN RESPONSE FACTOR F-BOX1* (*AFF1*) and our isolate *aff1-1* (Fig. 1h). The *aff1-1* mutant carries a C-to-T transition in the first exon of *AFF1*, resulting in the substitution of the Pro-93 with a Leu residue (Fig. 1h). The AFF1 protein contains an N-terminal F-box domain, a leucine rich repeat (LRR) region, and a C-terminal FBD motif (Fig. 1h).

Additional alleles and complementation analysis supported our hypothesis that the *At3g49150* mutation was causative in *aff1-1*. Three insertional alleles (Fig. 1h), which we named *aff1–2* (Salk_053818), *aff1–3* (Salk_083453), and *aff1–4* (Sail_427_G06) displayed ARF19 hypercondensation similar to *aff1-1* (Supplementary Figs. 1a and 2a). Moreover, we complemented *aff1-1* with a wild-type copy of *AFF1* (Supplementary Figs. 1b, 2a, 2b). These additional alleles and complementation lines confirm that the *At3g49150*/*AFF1* mutation is causative for the ARF19 hypercondensation observed in *aff1-1*.

### SCF^AFF1^ regulates ARF19 and ARF7 accumulation

**Fig. 2:**
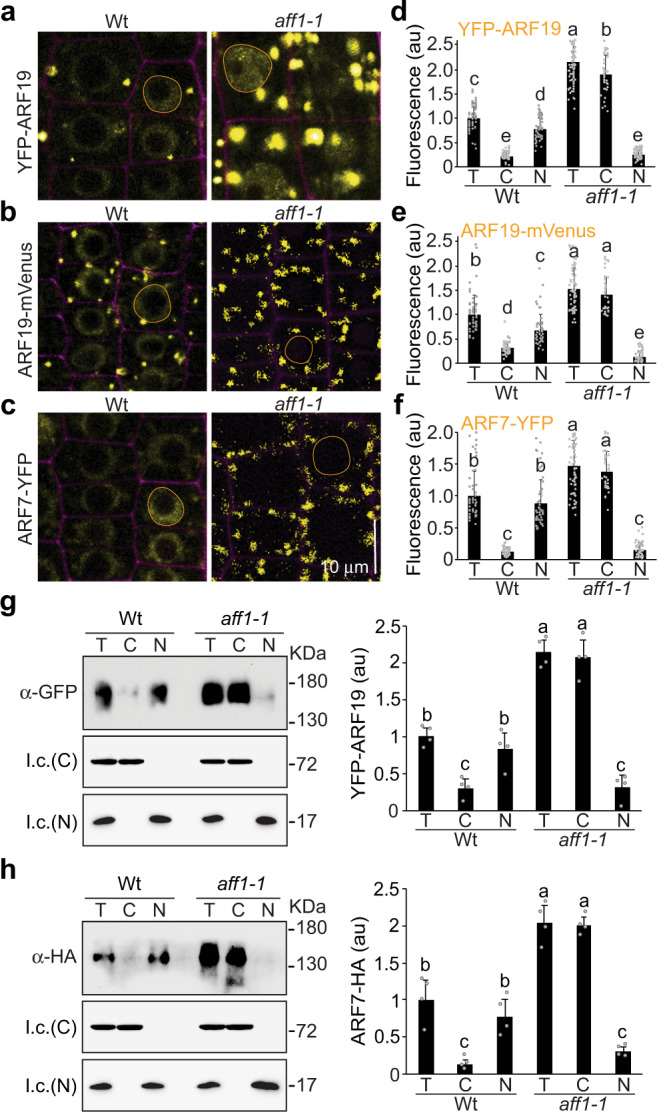
*AFF1* alters ARF19 and ARF7 nucleo-cytoplasmic partitioning. **a** Confocal images of root tip cells 3d-old Wt (Col-0) and *aff1-1* seedlings carrying *35* *S:YFP-ARF19*. **b** Confocal images of root tip cells 3d-old Wt (Col-0) and *aff1-1* seedlings carrying *pARF19:ARF19-mVenus*. **c** Confocal images of root tip cells 3d-old Wt (Col-0) and *aff1-1* seedlings carrying *pARF7:ARF7-YFP*. For each confocal image, the ARF signal is false-colored yellow with cell walls counterstained with propidium iodide (false-colored magenta). In each image a representative nucleus is circled with an orange line. Scale bar = 10 µm. **d**, **e**, **f** Quantification of subcellular fluorescence of 3d-old Wt (Col-0) and *aff1-1* seedlings carrying *35* *S:YFP-ARF19* (**d**)*, pARF19:ARF19-mVenus* (**e**), or *pARF7:ARF7-YFP* (**f**) *n* = 63, *n* = 52 and *n* = 52 independent cells were examined in **d**, **e** and **f**, respectively. Data are mean ± SD of three independent experiments and gray dots represent the individual values. Different letters indicate individual groups for multiple comparisons with significant differences (one-way ANOVA, Duncan, *p*  <  0.05). T (total), C (cytoplasmic), and N (nuclear). **g** Immunoblot analysis and quantification of YFP-ARF19 fractionation from 4d-old seedlings. **h** Immunoblot analysis and quantification of ARF7-HA fractionation from 4d-old seedlings. HSC70 and histone H3 served as loading controls (l.c.) for the cytosol (C) and the nucleus (N), respectively. Data in **g** and **h** are mean ± SD from four independent experiments and gray dots represent the individual values. Different letters indicate individual groups for multiple comparisons with significant differences (one-way ANOVA, Duncan, *p*  <  0.05). The source data in **d**, **e**, **f**, **g** and **h** are provided as a Source Data file.

Our previous work showed that ARF7 and ARF19 protein condensates are primarily localized to the cytoplasm whereas nuclear ARF7 and ARF19 are diffuse^2^. We therefore wanted to understand whether ARF nucleo-cytoplasmic partitioning is affected in *aff1*. Despite the increased numbers and sizes of ARF7 and ARF19 condensates, we were surprised to find that *aff1* displayed decreased nuclear accumulation of ARF19 and ARF7 when examined by microscopy (Fig. 2a–c). We were unable to separate ARF cytoplasmic condensates from nuclei using differential centrifugation because ARF condensates are dense and co-migrate with nuclei in these assays. Therefore, to quantify the partitioning of ARF proteins in the nucleus and the cytoplasm, we isolated nuclei from plant lysate using Concanavalin A-conjugated beads. Concanavalin A (lectin) binds specifically to the saccharide (mannosyl and glucosyl)-containing glycoproteins, such as glycosylated transmembrane proteins on the nuclear envelope, allowing for efficient nuclei isolation^6,7^. Whereas the majority of ARF19 (Fig. 2d, e, and g) and ARF7 (Fig. 2f and h) protein resided in the nuclei of wild-type root tip cells, the majority of these proteins were found in the cytoplasmic fractions of *aff1* root tip cells (Fig. 2d–h), suggesting that either ARF hypercondensation depletes the nuclear ARF fraction or that AFF1 plays dual roles in regulating ARF condensation and ARF nucleo-cytoplasmic partitioning.

**Fig. 3:**
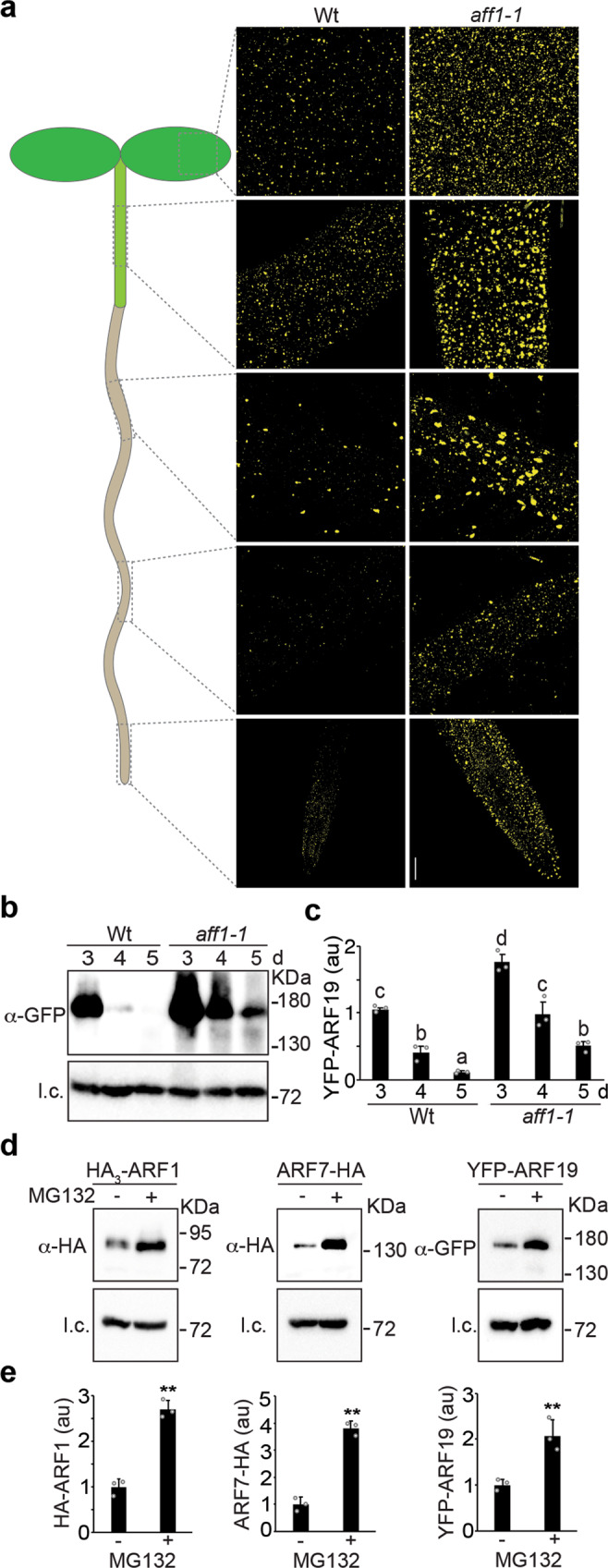
*AFF1* regulates ARF19 and ARF7 accumulation. **a** Confocal images of YFP-ARF19 fluorescence from various tissues of 3d-old wild type (Wt; Col-0) and *aff1-1* seedlings carrying *35* *S:YFP-ARF19* (false-colored yellow). Three independent experiments were performed with similar results. Scale bar = 50 µm. **b** Immunoblot analysis of 3d-, 4d-, or 5d-old wild type (Wt; Col-0) or *aff1-1* seedlings carrying *35* *S:YFP-ARF19*. Anti- GFP antibodies were used to detect YFP-ARF19, and anti-HSC70 antibodies were used to detect HSC70 (l.c.; loading control). **c** Quantification of YFP-ARF19 protein levels of 3d-, 4d-, or 5d-old wild type (Wt; Col-0) or *aff1-1* seedlings carrying *35* *S:YFP-ARF19*. Data are mean ± SD from three independent experiments and gray dots represent the individual values. Different letters indicate individual groups for multiple comparisons with significant differences (one-way ANOVA, Duncan, *p*  <  0.05). **d** Immunoblot analysis of HA_3_-ARF1, ARF7-HA, and YFP-ARF19 in seedlings treated with mock (DMSO) or MG132. Anti-HA antibodies were used to detect HA_3_-ARF1, ARF7-HA, anti-GFP antibodies were used to detect YFP-ARF19, and anti-HSC70 antibodies were used to detect HSC70 (l.c.; loading control). **e** Quantification of HA_3_-ARF1, ARF7-HA, and YFP-ARF19 accumulation in seedlings treated with mock (DMSO) or MG132. Data are mean ± SD from three independent experiments and the gray dots represent the individual values. The statistical significance was determined by a two-sided Student’s *t*-test (Paired two sample for means). *P* values = 0.00043 (HA_3_-ARF1), 0.0093 (ARF7-HA), 0.0019 (YFP-ARF19). **P < 0.01 when compared to the mock. The source data in (**b)**, (**c**), (**d**) and (**e**) are provided as a Source Data file.

*AFF1* is annotated as encoding an F-box protein, a type of E3 ubiquitin ligase that typically facilitates polyubiquitylation of targets to promote proteasomal degradation. Consistent with this role, we found that the *aff1* mutant displayed elevated YFP-ARF19 (Fig. 3a–c, and Supplementary Fig. 2) and ARF7-HA (Supplementary Fig. 2d) accumulation in every tissue examined (Fig. 3a) compared to wild type. We further found that HA_3_-ARF1, ARF7-HA, and YFP-ARF19 accumulation was elevated in samples treated with the 26 S proteasome inhibitor MG132 (Fig. 3d, e), consistent with these proteins being turned over by this protein degradation machinery.

**Fig. 4:**
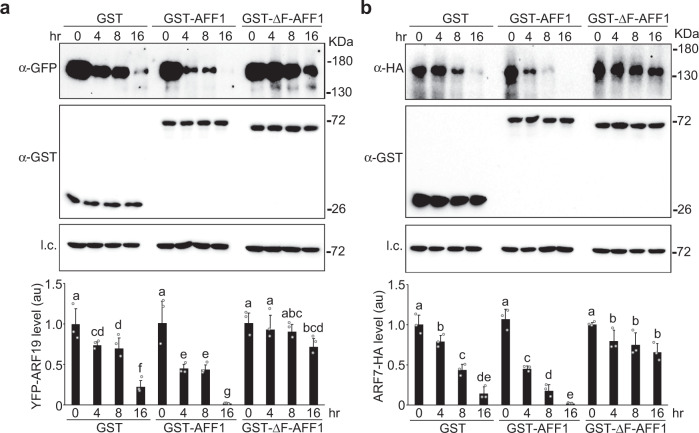
*AFF1* regulates ARF19 and ARF7 protein degradation. **a** In vitro YFP-ARF19 degradation. Plant lysate from *aff1-1 arf19-1 35* *S:YFP-ARF19* was incubated with GST, GST-AFF1, or GST-ΔF-box-AFF1 recombinant proteins for the indicated times. Immunoblot analysis (top) and quantification (bottom) of YFP-ARF19, GST, GST-AFF1, or GST-ΔF-box-AFF1 using the indicated antibodies. Anti-HSC70 used for loading control (l.c.). **b** In vitro ARF7-HA degradation. Plant lysate from *aff1-1 35* *S:ARF7-HA* was incubated with GST, GST-AFF1, or GST-ΔF-box-AFF1 recombinant proteins for the indicated times. Immunoblot analysis (top) and quantification (bottom) of ARF7-HA, GST, GST-AFF1, or GST-ΔF-box-AFF1 using the indicated antibodies. Anti-HSC70 used for loading control (l.c.). Data are mean ± SD of three independent experiments. Different letters indicate individual groups for multiple comparisons with significant differences (one-way ANOVA, Duncan, *p*  <  0.05) and gray dots represent the individual values. The source data in (**a**) and (**b**) are provided as a Source Data file.

In vitro YFP-ARF19 and ARF7-HA protein degradation assays showed that incubating plant lysate with GST-AFF1 recombinant protein increased ARF19 and ARF7 degradation compared to incubation with GST (Fig. 4). Conversely, incubation of plant lysate with GST-ΔF-box-AFF1, a truncation of AFF1 that should be unable to incorporate into an SCF complex but retain the ability to interact with substrates, reduced YFP-ARF19 and ARF7-HA degradation (Fig. 4), suggesting that this truncation protected ARF19 from endogenous degradation machinery. Thus, AFF1 regulates ARF19 and ARF7 protein accumulation.

**Fig. 5:**
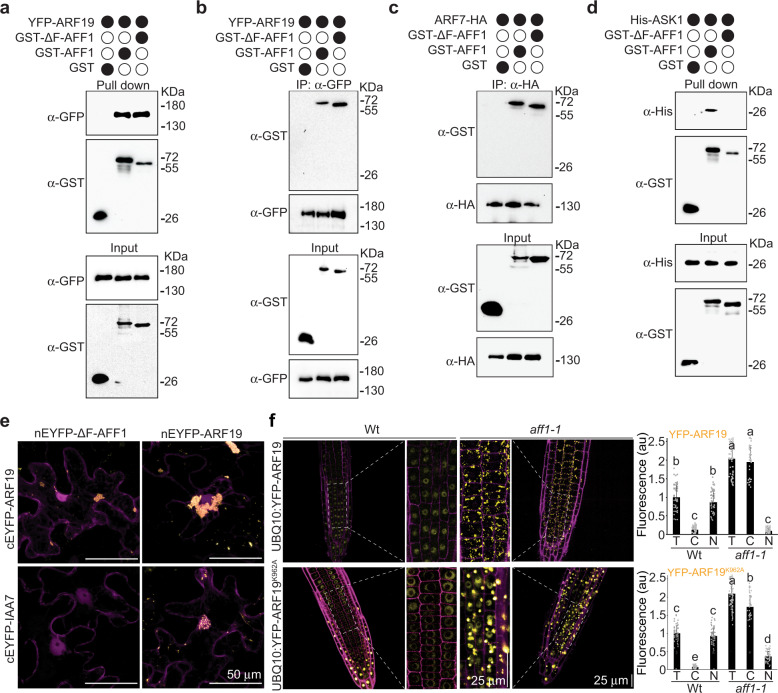
AFF1 interacts with ARF proteins and ASK1. **a** GST, GST-AFF1 or GST-ΔF-box-AFF1 recombinant proteins were incubated with *arf19-1 35* *S:YFP-ARF19* plant lysate. Pull-down fractions and inputs were examined by immunoblot analysis using anti-GFP or anti-GST antibodies. **b** GST, GST-AFF1 or GST-ΔF-box-AFF1 were incubated with *arf19-1 35* *S:YFP-ARF19* plant lysate prior to immunoprecipitation with anti-GFP antibody. Immunoprecipitates and inputs were examined by immunoblot analysis using anti-GFP or anti-GST antibodies. **c** GST, GST-AFF1 or GST-ΔF-box-AFF1 were incubated with *35* *S:ARF7-HA* plant lysate prior to immunoprecipitation with anti-HA antibody. Immunoprecipitates and inputs were examined by immunoblot analysis using anti-HA or anti-GST antibodies. **d** GST, GST-AFF1 or GST-ΔF-box-AFF1 were incubated with His-ASK1 prior to pull down. Pull-down fractions and inputs were examined by immunoblot analysis using anti-His or anti-GST antibodies. **e** Bimolecular fluorescence complementation (BiFC; yellow) assays were used to analyze protein interactions between nEYFP-ΔF-box-AFF1 and cEYFP-ARF19, nEYFP-ΔF-box-AFF1 and cEYFP-IAA7, nEYFP-ARF19 and cEYFP-ARF19, or nEYFP-ARF19 and cEYFP-IAA7. The nuclear marker WPP-mCherry (magenta) was co-expressed to determine nuclear signal. Scale bar = 50 µm. See Supplementary Fig. 3 for extended data. **f** Left, confocal images of 3d-old wild type (Wt; Col-0) or *aff1-1* seedlings carrying *UBQ10:YFP-ARF19* or *UBQ10:YFP-ARF19*^*K962A*^ (false-colored yellow) with cell walls counterstained with propidium iodide (false-colored magenta). Scale bar = 25 µm. Right, quantification of subcellular fluorescence. T (total), C (cytoplasmic), and N (nuclear). See Supplementary Fig. 4 for images from additional regions of the root. Three independent experiments were performed on (**a**), (**b**), (**c**), (**d**), (**e**) and (**f**) with similar results. *n* = 56 (*ARF19*) and *n* = 54 (*ARF19*^*K962A*^) independent cells were examined in (f). Data in (f) are mean ± SD and gray dots represent the individual values. Different letters indicate individual groups for multiple comparisons with significant differences (one-way ANOVA, Duncan, *p*  <  0.05). The source data in (**a**), (**b**), (**c**), (**d**) and (**f**) are provided as a Source Data file.

Because AFF1 is annotated as an F-box protein and affects ARF19 and ARF7 protein degradation, we explored whether AFF1 could be incorporated in an SCF complex and whether this potential SCF^AFF1^ complex could directly target ARF19 and ARF7 to the proteasome. First, we assessed the interaction between AFF1 and ARF proteins. Although we were unable to heterologously express full-length ARF19 protein; heterologously-expressed GST-AFF1 and GST-ΔF-box-AFF1 pulled down YFP-ARF19 protein from plant lysate (Fig. 5a). Further, YFP-ARF19 and ARF7-HA purified from plant lysate could interact with GST-AFF1 and GST-ΔF-box-AFF1 recombinant proteins, but not with GST (Fig. 5a, b). Thus, AFF1 interacts with both ARF19 and its close homolog ARF7. In a bimolecular fluorescence complementation (BiFC) assay, we found that AFF1 interacted with ARF19 protein, but failed to interact with the Aux/IAA repressor protein IAA7, a protein in the auxin signaling pathway that shares a similar PB1 domain as found in ARFs (Fig. 5e and Supplementary Fig. 3). Moreover, AFF1-ARF19 and ARF19-ARF19 interactions appeared to occur in cytoplasmic condensates whereas ARF19 and IAA7 appeared to interact primarily in the nucleus (Fig. 5e and Supplementary Fig. 3). Although the BiFC system artificially overexpresses proteins, these data are consistent with the possibility that AFF1 targets the cytoplasmic fraction of ARF proteins.

Next, we tested whether AFF1 could directly interact with ARABIDOPSIS SKP1-LIKE (ASK1), an adaptor connecting the subunit CULLIN 1 (CUL1) in the SCF complex^8^. In pull-down experiments, GST-AFF1, but not GST-ΔF-box-AFF1, interacted with heterologously-expressed His-ASK1 (Fig. 5d), suggesting that AFF1 incorporates into an SCF E3 ubiquitin ligase complex to form SCF^AFF1^. The direct interaction of AFF1 with ARF7 and ARF19 proteins, combined with our data that AFF1 regulates ARF19 and ARF7 accumulation, leads to a model in which SCF^AFF1^ regulates ARF19 and ARF7 accumulation.

ARF19 condensate formation relies both on oligomerization through its Phox and Bem1p (PB1) domain and presence of its intrinsically disordered middle region^2^. In wild-type seedlings, wild-type ARF19 protein displays predominantly nuclear localization in root tips, when expressed behind either the native promoter or behind the *UBQ10* constitutive promoter (Figs. 1c, 2a, 2b, 5f, Supplementary Fig. 4). However, in parts of the root no longer undergoing active growth (i.e., the upper root cells), ARF19 protein is found in cytoplasmic condensates in wild type^2^. In contast, ARF19 protein is found in cytoplamsic condensates in all examined *aff1* root cells (Figs. 1b, 1c, 2a, 2b, 5f, Supplementary Fig. 4). Mutation of the conserved lysine (K962) in the PB1 domain disrupts PB1-mediated oligomerization and is sufficient to disrupt ARF19 condensation, resulting in nuclear ARF19^K962A^ localization in all cell types, including those in which ARF19 is found primarily in cytoplasmic condensates^2^. ARF19 PB1 domain oligomerization is thought to drive up the local concentration of ARF19 protein to overcome the biophysical threshold at which this protein enters a two-phase regime, or undergoes condensation. Unlike in wild type, ARF19^K962A^ forms condensates in *aff1* (Fig. 5f), suggesting that hyperaccumulation of this protein in the *aff1* mutant background overcomes the requirement of PB1 oligomerization to drive concentration-dependent ARF condensation.

Taken together, our results suggest roles for SCF^AFF1^ in regulating ARF7 and ARF19 protein accumulation, condensation, and nucleo-cytoplasmic partitioning.

### *aff1* displays developmental defects and altered auxin responsiveness

**Fig. 6:**
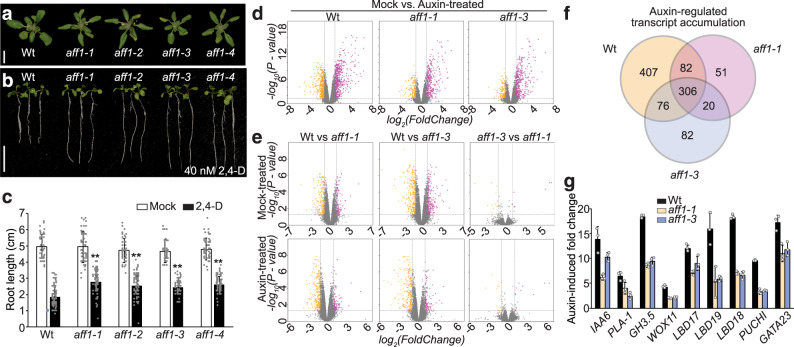
*aff1* exhibits developmental defects and attenuated auxin responsiveness. **a** Photograph of 22d-old wild type (Wt; Col-0), *aff1-1*, *aff1–2*, *aff1–3*, and *aff1–4* plants. Scale bar = 1 cm. **b** Photograph of 9d-old wild type (Wt; Col-0), *aff1-1*, *aff1–2*, *aff1–3*, and *aff1–4* seedlings grown on media supplemented with 40 nM 2,4-D. Scale bar = 1 cm. **c** Mean primary root lengths of 9d-old wild type (Wt; Col-0), *aff1-1*, *aff1-2*, *aff1–3*, and *aff1–4* seedlings vertically grown on media supplemented with mock (EtOH) or 40 nM 2,4-D. *n* = 80 biologically independent seedlings were examined. Data are mean ± SD from three independent experiments and gray dots represent the individual values. The statistical significance was determined by a two-sided Student’s *t*-test (Paired two sample for means). *P* values = 0.9815 (*aff1-1_*mock*)*, 0.3943 (*aff1-2_*mock*)*, 0.1653 (*aff1–3_*mock*)*, 0.8700 (*aff1–4_*mock*)*, 7.50E-17 (*aff1-1_*2,4-D), 1.315E-11 (*aff1-2_*2,4-D), 4.355E-12 (*aff1-3_*2,4-D), and 7.405E-13 (*aff1-4_*2,4-D). **P < 0.01 when compared to Wt. **d** Volcano plots displaying pairwise transcript accumulation differences after two hours of Mock (EtOH) or auxin (10 μM IAA) treatment in wild type (Wt; Col-0), *aff1-1*, and *aff1-3*. **e** Volcano plots displaying pairwise transcript accumulation differences between wild type (Wt; Col-0) and *aff1-1*, Wt and *aff1-3*, or *aff1-1* and *aff1–3* after 2h treatment with Mock (EtOH) or Auxin (10 μM IAA). FDR ≤ 0.01. **f** Venn diagrams showing the number of genes that are overlap between the datasets of differentially expressed genes (FDR < 0.01). **g** Relative transcript abundance (±SD, *n* = 3) of auxin response targets in wild type (Wt; Col-0), *aff1-1* and *aff1–3* with or without 10 μM IAA treatment for 2 h. Data are mean ± SD and gray dots represent the individual values. The source data in (**c**) and (**g**) are provided as a Source Data file. See Supplementary Fig. 6 for RNA Sequencing (RNA-seq) quality assessments.

Morphometric analysis of *aff1* alleles revealed elongated and downward-curled leaves (Fig. 6a and Supplementary Fig. 5), a phenotype often found in mutants defective in auxin signal transduction. In addition, *aff1* mutants displayed resistance to the inhibitory effects of the synthetic auxin 2,4-D on root elongation (Fig. 6b, c). These morphological phenotypes suggest that auxin responses are dampened in *aff1* mutants.

We examined global auxin-responsive transcript accumulation in wild type, *aff1-1* and *aff1–3* (Fig. 6d–f). Both alleles were mildly less responsive than wild type, with multiple transcripts displaying attenuated auxin responsiveness (Fig. 6e, f). Further, examination of direct targets of ARF19, such as *LBD17*, *LBD19*, *LBD18*, *PUCHI*, and *GATA23*, revealed decreased auxin-induced transcript accumulation in *aff1* mutants compared to wild type (Fig. 6g). Consistent with their morphological phenotypes (Fig. 6a and Supplementary Fig. 5) and decreased ARF7 and ARF19 nuclear accumulation (Fig. 2), *aff1* mutants displayed decreased auxin-responsive transcript accumulation in these analyses. Thus, our data suggest that the attenuated auxin responses observed in *aff1* are caused by increased ARF7 and ARF19 condensation and decreased ARF7 and ARF19 protein accumulation.

**Fig. 7:**
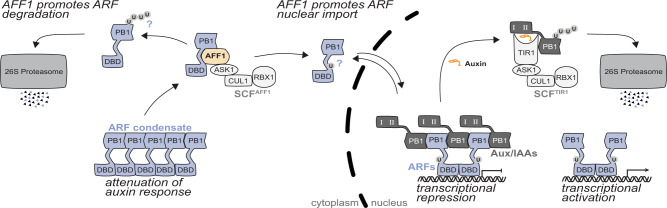
A proposed model for the SCF^AFF1^ role in regulating ARF condensation and nucleo-cytoplasmic partitioning. SCF^AFF1^ directly interacts with ARF19 and ARF7, suggesting that these transcription factors, and perhaps additional ARFs, are substrates of this putative E3 ubiquitin ligase. Further, the distinct *aff1* mutant effects on ARF protein accumulation/condensation and localization, along with the identity of AFF1 as an E3 ubiquitin ligase, raises the possibility that distinct ubiquitin moieties promote these distinct fates of the ARF transcription factors, both of which may be mediated by SCF^AFF1^.

Overall, our data support a model (Fig. 7) in which SCF^AFF1^ modulates ARF protein accumulation, condensation, and nucleo-cytoplasmic partitioning to regulate auxin responsiveness to affect plant growth and development.

## Discussion

The ARF7 and ARF19 transcription factors promote auxin response and drive several aspects of plant development^3^. Their tissue-specific competence to facilitate auxin transcriptional responses is regulated by protein condensation^2^. Here, we identify SCF^AFF1^ roles in regulating ARF7 and ARF19 condensation to modulate auxin responses and plant development. In *aff1*, ARF7 and ARF19 levels are reduced in root meristem nuclei and are primarily localized to cytoplasmic condensates. Reduction in nuclear ARF7 and ARF19 is accompanied by reduced auxin transcriptional responses and morphological defects.

AFF1 is an F-box type E3 ubiquitin ligase. Ubiquitin ligases facilitate posttranslational modification with a ubiquitin moiety to a target protein. Ubiquitin can be attached to target proteins as a single event (monoubiqitination) or as successive events to result in a polyubiquitin chain. Further, these ubiquitins can be connected through specific isopeptide bonds that result in distinct cellular outcomes^9^. ARF7 and ARF19 display increased protein accumulation in *aff1* and in vitro ARF7 and ARF19 protein degradation assays suggest that SCF^AFF1^ promotes degradation of these transcription factors. Because AFF1 is an E3 ubiquitin ligase that directly interacts with ARF7 and ARF19, SCF^AFF1^ effects on ARF protein stability is likely through polyubiquitylation and subsequent proteasomal degradation of these proteins. Further, because protein condensation is a concentration-dependent process^4^, the elevated ARF condensation in *aff1* could be driven by a simple ARF concentration change or by a more complex mechanism involving ARF ubiquitylation. In addition, it is also possible that AFF1 interaction with ARF7 and ARF19 could alter their condensation via a mechanism unrelated to ARF protein accumulation. Conversely, elevated ARF7 and ARF19 protein levels and increased condensation do not adequately explain the nuclear depletion of these transcription factors in the *aff1* mutant. The most plausible explanation for this data is that SCF^AFF1^ mediates monoubiquitylation, or perhaps a ubiquitin chain that does not promote degradation, of ARF7 and ARF19 that affects its nucleo-cytoplasmic partitioning, possibly by blocking a nuclear export signal^10^ similar to what is seen in transcription factors such as p53^11^.

ARF transcription factors are divided into three ancient clades - Class-A, B, and C ARFs; Class-A ARFs are generally thought to be transcriptional activators whereas Class-B and C ARFs are generally thought to repress transcription^12^. ARF7 and ARF19 are two class-A ARFs that are both regulated by the proteasome and directly interact with AFF1. Similarly, the class-A ARF6^13,14^ and ARF8^13^, the Class-B ARF1^15^, ARF2^16^, and the Class-C ARF17^13^ also undergo proteasome-dependent degradation. Whether these protein stabilities are regulated through the activity of SCF^AFF1^ or through another mechanism remains unknown. Indeed, ARF19 is not fully stabilized in the *aff1* mutant, suggesting that additional mechanisms regulate the stability of this transcription factor. In addition, stability of the Class-B ARF1 is proteasome-dependent and its accumulation is not altered in the *cul1* mutant background, suggesting that ARF1 proteasomal degradation is via an alternative set of machinery^15^. Thus, it seems likely that multiple mechanisms exist to regulate ARF protein accumulation. Further, we have not yet identified the ARF19 degron. However, ARF proteins that lack the PB1 domain (ARF17)^13^, or are truncated to lack the DNA binding domain (ARF1)^15^ are degraded in a proteasome-dependent manner. These findings raise the possibility that the ARF degron might lie within the intrinsically disordered middle region.

In summary, our genetic and biochemical evidence suggest SCF^AFF1^ promotes ARF7 and ARF19 accumulation, condensation, and nucleo-cytoplasmic partitioning to regulate auxin responses, providing new insight into the mechanisms behind the complex web of auxin-regulated responses and opening new paths of investigation into auxin biology.

## Methods

### Plant materials and phenotypic assays

All *Arabidopsis thaliana* lines were in the Columbia (Col-0) background, which was used as the wild type (Wt) in all experiments. For phenotypic assays, seeds were surface sterilized for 15 min with 20% (v/v) bleach and 0.01% (v/v) Triton X-100, then rinsed four times with sterile water. Sterilized seeds were suspended in 0.1% (w/v) agar and then stratified for 2 d at 4 °C to promote uniform germination. After stratification, seeds were plated on plant nutrient (PN) media solidified with 0.6% (w/v) agar and supplemented with 0.5% (w/v) sucrose (PNS) at 22 °C under continuous illumination.

To analyze the leaf phenotypes of Wt and mutants, seeds were directly germinated in the soil. Images were taken after 22 d of growth at 22 °C under continuous illumination. To examine root elongation in Wt and mutants, root lengths were measured from seedlings grown on media supplemented with mock (EtOH) or the indicated concentration of 2,4-D after 9 d of vertical growth at 22 °C under continuous illumination.

### Vector construction and plant transformation

To create *arf19-1 35* *S:YFP-ARF19*, the coding sequence of ARF19 was PCR amplified from cDNA using Pfx Platinum (Life Technologies). The resultant PCR product was cloned into pENTR/D-TOPO (Life Technologies) to create pENTR-ARF19. The pENTR-ARF19 vector was recombined into the pEarleyGate104 plasmid^17^ using LR Clonase (Invitrogen) to create 35 S:YFP-ARF19. Recombinant plasmid was transformed into *Agrobacterium tumefaciens* strain GV3101^18^, and then transformed into *arf19-1* mutant plants via the floral dip method. Transformants were selected selection on plant nutrient media supplemented with 10 μg/mL Basta. Subsequent generations were tested to identify lines homozygous for the transgene.

To create the rescue line *aff1-1 35* *S:AFF1genomic*, the genomic sequence of *AFF1* was cloned into pENTR/D-TOPO to create pENTR-AFF1g. The pENTR-AFF1g vector was recombined into the pMDC32 plasmid using LR Clonase. Recombinant plasmid was transformed into GV3101 and then used to transform into the *aff1-1* mutant via the floral dip method. Transformants were selected by plating on media supplemented with 25 μg/mL hygromycin. Subsequent generations were tested to identify lines homozygous for the transgene.

The coding sequence of the *AFF1* was synthesized and cloned into the pENTR/D-TOPO vector. The CDS of *AFF1* and ΔF-box-AFF1 were PCR amplified from the pENTR/D-TOPO vector, and then cloned into the *Bam*HI and *Sal*I sites of the pGEX4T-1 (Amersham Biosciences) to generate pGEX4T-AFF1 and GEX4T-ΔF-box-AFF1 expression vectors to express GST- AFF1 and GST-ΔF-box-AFF1, respectively. The coding sequence of *ASK1* was PCR amplified from Arabidopsis cDNA, and then cloned into the *Bam*HI and *Hind*III sites of pET28a (Novagen) to make pET28-ASK1 expression vectors to express His-ASK1.

To create the bimolecular fluorescence complementation (BiFC) expression vectors, the pENTR-ΔF-box-AFF1, pENTR-TOPO-IAA7 and pENTR-ARF19 were recombined into the pSITE-nEYFP-C1 or pSITE-cEYFP-N1 (from ABRC) using LR Clonase. To create nuclear marker WPP-mCherry, the coding sequence of the WPP domain (amino acids 1–111) of the gene *RanGAP* (*At3g63130*) fused with mCherry was synthesized and cloned into the pENTR/D-TOPO vector. The *ACT2* promoter was cloned into pENTR-WPP-mCherry using K*pn*I and X*ho*I restriction sites. Then the pENTR-ACT2-WPP-mCherry vector was recombined into the pMDC99 plasmid using LR Clonase to create pMDC99-ACT2-WPP-mCherry.

All primers used for plasmid construction are listed in Supplementary Table 1.

### EMS mutagenesis and mutant identification

To perform the mutant screen, seeds of *arf19-1 35* *S:YFP-ARF19* were mutagenized by incubation with 0.24% (v/v) ethyl methanesulfonate (EMS) for 16 hr, and then rinsed four times with sterile water. Mutagenized seeds were suspended in 0.1% (w/v) agar, then directly planted to soil. M_2_ seeds from were plated on PNS media and grown for 8 d at 22 °C under continuous illumination. Candidate mutants displaying elevated YFP-ARF19 condensation, as viewed through a fluorescence dissecting microscope, were transferred to soil, grown at 22 °C under continuous illumination, and allowed to self-fertilize.

A whole genome sequencing of bulk segregants approach was used to identify the causative mutation in DH8 mutant, which was described previously^5^. We back-crossed DH8 three times into the wild type (Col-0) background. Nearly 500 progeny of a BC_3_F_3_ population were genotyped using PCR analysis to separate the *aff1-1* lesion from nearby mutations, particularly the mutation in nearby *RDR6* (*At3g49500*). The genotyping primers designed by dCAPS are listed in Supplementary Table 1.

### Confocal microscopy

For confocal images of plant lines, seedlings were mounted in water under a coverslip and imaged though a 40x lens on a Leica TCS SP8 confocal microscope.

### Condensates fusion and circularity analysis

The fusion and circularity analysis were performed on condensates from the *pUBQ10:YFP-ARF19* (Upper root) and *aff1-1 pUBQ10:YFP-ARF19* (Upper root) lines using a Leica SP8 confocal microscope. All imaging was carried out using a PMT detector and a 40 × water immersion objective. Imaging used a 512 × 512 format and a scan speed of 400 Hz. For image acquisition, 300 images were captured at 1 s intervals for a total of 6 min. The images were loaded into FIJI (ImageJ) and use the “Process” tool to set up the subtract background. Then the “Image” tool was used to adjust the threshold. Each individual condensate was analyzed by “Analyze particles” to determine the circularity.

### Fluorescence recovery after photobleaching (FRAP)

FRAP experiments were performed on condensates from the pUBQ10:YFP-ARF19 (Upper root) and *aff1-1* pUBQ10:YFP-ARF19 (Upper root and root tip) lines using a Leica SP8 confocal microscope. All imaging was carried out using a PMT detector and a 40 × water immersion objective under the Leica FRAP module. Imaging used a 512 × 512 format and a scan speed of 400 Hz. Method of bleaching were set as follows: fly mode – off, zoom in – on, change bleach format – off, set background to zero – on, delete bleach images after scan – off, Use laser settings for all ROIs – on. For photobleaching, the 488 nm, 514 nm and 552 nm were set to 100% power. Two pre-bleach images were acquired followed by the photobleaching and then 100 postbleach images were captured at 5.17 s intervals. After image acquisition, data was imported into FIJI (ImageJ) for further analysis.

### Immunoblot analysis

Immunoblot analysis was performed as described previously^19^. Total cellular proteins were prepared by grinding plant materials in liquid nitrogen and then extracted in grinding buffer (50 mM Tris-HCl, pH 8.0, 150 mM NaCl, 1% (v/v) Nonidet P-40, 0.5% (w/v) sodium deoxycholate, 0.1% (w/v) SDS, 1 mM phenylmethylsulfonyl fluoride and 1% (v/v) protease inhibitors cocktail (Sigma-Aldrich, P9599). After heating at 100 °C for 10 min, the samples were then subjected to SDS-polyacrylamide gel electrophoresis. After the run, proteins were transferred onto a nitrocellulose membrane, and then detected with 1:5000 of the indicated primary antibody (Anti-Histone H3, Agrisera AS10–710; anti-HA 3F10, Sigma 11867423001; anti-His H-15, Santa Cruz sc-803; anti-HSC70 5B7, Enzo ADI-SPA-817-D; anti-GST B-14; Santa Cruz sc-138; anti-GFP-HRP 1E10H7; Fisher 50-553-599). The blot was incubated with a secondary antibody (goat anti-mouse IgG-HRP or goat anti-rabbit IgG-HRP, Santa Cruz Biotechnology) at 1:5000 dilution. The signal was detected using a WesternBright ECL HRP substrate kit (Advansta) according to the manufacturer’s instructions. Arabidopsis HSC70 was used as a loading control. The target bands and loading control bands were quantified using ImageJ and the mean values of 3–5 independent experiments were presented with statistical analysis (one-way ANOVA or Student’s *t*-test) of significant differences when applicable.

For MG132 (Sigma-Aldrich, M8699) treatment, *UBQ10:HA*_*3*_*-ARF1*, *35* *S:ARF7-HA*, or *arf19-1 35* *S:YFP-ARF19* were grown on PNS media for 3 d at 22 °C under continuous illumination. Seedlings were then transferred to liquid PN supplemented with either DMSO (Mock) or 50 μM MG132 for 16 h. Then the samples were collected for following immunoblot analysis. Three independent experiments were used for quantitative analysis.

### Cell fractionation

Cytoplasmic and nuclei were isolated as previously described with modifications^20^. Around 0.5 g of 4d-old seedlings (*35S:ARF7-HA*, *aff1-1 35S:ARF7-HA*, *35* *S:YFP-ARF19* and *aff1-1 35* *S:YFP-ARF19*) were fresh frozen in liquid nitrogen and ground into fine power. Cells were resuspended in 2 ml lysis buffer (20 mM Tris-HCl pH 7.4, 25% glycerol, 20 mM KCl, 2 mM EDTA, 2.5 mM MgCl_2_, 250 mM sucrose, 1 mM DTT, 50 μM MG132, 1 mM PMSF and 1 × Roche protease inhibitor cocktail). The lysate was sequentially filtered through 70-μm and 40-μm cell strainers to remove cell debris and flow-through was taken as the total lysate. Then the lysate was centrifuged at 1500 g for 15 min at 4 °C and supernatant was collected as partial cytoplasmic fraction. The pellet was resuspended in 1 ml lysis buffer and incubated with Concanavalin A coated magnetic beads (Polysciences, Inc. #86057) for 2 h at 4 °C, then the nuclei will bind to the beads. After that, the nuclei binding beads were gently washed 3 times with the wash buffer (20 mM Tris-HCl, pH 7.4, 25% glycerol, 250 mM NaCl, 2.5 mM MgCl_2_, and 0.15% Triton X-100, 1 mM DTT, 1 mM PMSF and 1 × of Roche protease inhibitor cocktail) and resuspended in lysis buffer as the nuclear fraction. The left lysate was centrifuged at 1500 g for 15 min at 4 °C and the pellet was collected to the previous partial cytoplasmic fraction as the total cytoplasmic fraction. Protein samples were then boiled at 100 °C for 5 min, and run on the SDS-PAGE for further immunoblot analysis. Cell fractionation was confirmed by antibodies against cytoplasmic marker HSC70 (α-HSC70) and nuclear marker histone H3 (α-H3).

### Bimolecular fluorescence complementation (BiFC) assay

Bimolecular fluorescence complementation (BiFC) experiments were conducted as previously described^21^. Briefly, the resulting binary expression vectors were transformed into *Agrobacterium* strain GV3101. Collected cells were washed and resuspended to OD_600_ of approximately 1.0 with the infiltration solution (10 mM MES, pH 5.6, 10 mM MgCl_2_, and 1 mM acetosyringone). *Agrobacterial* cells carrying various expression vectors with the p19 strain were co-infiltrated into 3-week-old *Nicotiana benthamiana* leaves. Empty vectors were used as negative controls. After the infiltration, plants were placed at 22 °C for 3 d and the YFP and mCherry fluorescent signals were detected using Leica TCS SP8 confocal microscope. The experiment was repeated three times with independent biological replicates.

### Pull-down assays

Protein pull-down assays were performed as described^22^ with minor modifications. To analyze the interaction between ARF19 or ARF7 with AFF1, plant samples from *arf19-1 35* *S:YFP-ARF19* or *35* *S:ARF7-HA* were ground in liquid nitrogen, and then extracted in grinding buffer (50 mM Tris-HCl, pH 7.5, 150 mM NaCl, 10 mM MgCl_2_, 10% (v/v) glycerol, 0.1% (w/v) Nonidet P-40, 1 mM phenylmethylsulfonyl fluoride, 1% (v/v) protease inhibitors cocktail (Sigma-Aldrich, P9599) and 10 μM MG132. Purified GST, GST-AFF1, and GST-ΔF-box-AFF1 proteins were immobilized on GST beads (Glutathione Agarose; ThermoFisher). Immobilized agarose beads containing 2 μg GST, GST-AFF1, or GST-ΔF-box-AFF1 fusion proteins were mixed with 1–2 mg total cellular proteins from *arf19-1 35* *S:YFP-ARF19* or *35* *S:ARF7-HA* at 4 °C for 2 hr. The beads were collected by centrifugation and then washed six times with washing buffer (10 mM phosphate buffer saline, pH 7.4, 150 mM NaCl, 0.2% (v/v) Triton X-100 and 1 mM phenylmethanesulfonyl fluoride) at 4 °C. The beads were resuspended in SDS-polyacrylamide gel electrophoresis sample buffer and then analyzed by immunoblot.

To detect the interaction between GST-AFF1 and His-ASK1, immobilized agarose beads containing 2 μg GST, GST-AFF1, or GST-ΔF-box-AFF1 fusion proteins were mixed with 2 μg His-ASK1 at 4 °C for 2 hr. The beads were collected after washing six times to do the SDS-polyacrylamide gel electrophoresis, and then analyzed by immunoblot.

### Co-immunoprecipitation assay

The Co-IP experiments were performed according to previously described methods^23^ with minor modifications. To prepare total cellular proteins, plant samples were grinded in liquid nitrogen, and then extracted in grinding buffer (50 mM Tris-HCl, pH 7.5, 150 mM NaCl, 10 mM MgCl_2_, 10% (v/v) glycerol, 0.1% (v/v) Nonidet P-40, 1 mM phenylmethylsulfonyl fluoride, 1% (v/v) protease inhibitors cocktail (Sigma-Aldrich, P9599) and 10 μM MG132. The extracts containing 1.0–2.0 mg total cellular proteins were incubated with 10 μl anti-GFP or anti-HA antibodies for 1 hr at 4 °C with gentle shaking. After that, the Dynabeads Protein G (50 μl, ThermoFisher) were added and mixed with 2 μg GST, GST-AFF1, or GST-ΔF-box-AFF1 fusion proteins for an additional 2 hr at 4 °C. The immunoprecipitates were washed six times with 1 ml washing buffer (grinding buffer without MG132) and then used for immunoblot.

### In vitro turnover assay

The analysis of YFP-ARF19 and ARF7-HA protein degradation in vitro was performed as described methods with minor modifications^19^. In brief, total protein extracts were prepared from 3d-old parental line *arf19-1 35* *S:YFP-ARF19* or *aff1-1 35* *S:ARF7-HA* grown in PNS medium using ice-cold extraction buffer (50 mM Tris-HCl, pH 7.5, 150 mM NaCl, 0.01% (v/v) Triton X-100 and 1 mM phenylmethanesulfonyl fluoride). The crude extracts (1 mg proteins) were mixed with 2 μg of purified GST, GST-AFF1, or GST-ΔF-box-AFF1 recombinant proteins in a total volume of 600 μl. The mixture was incubated at 4 °C with gentle agitation and 100 μl of each sample was collected at the indicated time points and then analyzed by immunoblotting.

### Quantitative reverse transcription-PCR (qRT-PCR)

Total RNA was prepared using the RNeasy Plant Mini Kit (Qiagen). Quantitative reverse transcription-PCR (qRT-PCR) was performed using the iTaq™ Universal SYBR® Green Supermix (Bio-Rad) according to the manufacturer’s instructions. The reactions were run in a CFX96 REAL-Time PCR Detection System (Bio-Rad). The relative expression level of the target genes was analyzed with the delta-delta Ct method and normalized to the expression level of *ACT7*. All of the experiments were repeated for at least twice (two biological repeats with three technical repeats for each experiment). The primers used for qRT-PCR are listed in Supplementary Table 1.

### RNA-Seq

RNA-Seq experiment were performed according to previously described methods^2^. Col-0 (Wt), *aff1-1*, and *aff1-3* (Salk_083453) were grown on PNS media for 4 d at 22 °C under continuous illumination. Seedlings were then transferred to liquid PN supplemented with either ethanol (Mock) or 10 μM IAA for 2 h. Three repeated treatments were carried out for each line. Total RNA was isolated using the RNeasy Plant Mini Kit (Qiagen). RNA samples were then sequenced using the Epicentre Ribo-Zero Gold system according to manufacturer’s protocol, indexed, pooled, and sequenced across three 1x50bp lanes on a single flow-cell on an Illumina HiSeq 3000. RNA-seq reads were demultiplexed and aligned to the Ensembl release 23 (TAIR 10) top-level assembly with STAR version 2.0.4b. Gene counts were derived from the number of uniquely aligned unambiguous reads by Subread:featureCount version 1.4.5. Sequencing performance was assessed for total number of aligned reads, total number of uniquely aligned reads, and genes detected.

All gene counts were imported into the R/Bioconductor package EdgeR and TMM normalization size factors were calculated to adjust for samples for differences in library size. Ribosomal genes and genes not expressed in any sample greater than one count-per-million were excluded from further analysis. In addition, genes not expressed in at least 2 out of the 3 samples were not considered for downstream analysis. The TMM size factors and the matrix of counts were then imported into R/Bioconductor package Limma. Performance of the samples was assessed with a Spearman correlation matrix and Multi-Dimensional Scaling plot (Supplementary Fig. 6a, b). Weighted likelihoods based on the observed mean-variance relationship of every gene and sample were then calculated for all samples with the voomWithQualityWeights function and gene performance was assessed with plots of residual standard deviation of every gene to their average log-count with a robustly fitted trend line of the residuals (Supplementary Fig. 6c). A generalized linear model was then created to test for gene level differential expression and the results were filtered for only those genes with Benjamini-Hochberg false discovery rate adjusted *p* values less than or equal to 0.05.

For volcano plots and heat maps, data was imported using the Pandas python package. For volcano plots, the bioinfokit Python package was used (visuz.gene_exp.volcano), and vertical lines represent the LFC of 1.5 and the horizontal lines represent adjusted p-values of 0.05. For heat maps, the Python package Seaborn was used (seaborn.clustermap) with a custom color map using matplotlib.colors.

The RNASeq data discussed in this publication have been deposited in NCBI’s Gene Expression Omnibus and are accessible through GEO Series accession number GSE172353.

### NanoStrings Analysis

NanoStrings analysis experiment were performed according to previously described methods^2^. Col-0 (Wt), *aff1-1*, and *aff1–3* (Salk_083453) were grown on PNS media for 4 d at 22 °C under continuous illumination. Seedlings were then transferred to liquid PN supplemented with either ethanol (Mock) or 10 μM IAA for 2 h. Three repeated treatments were carried out for each line. Total RNA was isolated using the RNeasy Plant Mini Kit (Qiagen). NanoString nCounter analysis was performed using 80 ng total RNA and carried out using the nCounter Digital Analyzer (NanoStrings Technologies; Seattle, WA) at the McDonnell Genome Institute at Washington University in St. Louis. In addition to 8 negative-control and 6 positive-control probes, two genes *TUB4* (*At5g44340*) and *PP2C* (*At1g13320*) were used as references for normalization. Data was analyzed using the nSolver Analysis software.

### Reporting summary

Further information on research design is available in the Nature Research Reporting Summary linked to this article.

## Supplementary information

Supplementary Information

Reporting Summary

## Data Availability

The RNA-seq data discussed in this publication have been deposited in NCBI’s Gene Expression Omnibus and are accessible through GEO Series accession number GEO: GSE172353. Source data are provided with this paper.

## Source data

Source Data
