# Supplementary material for: Regulation of AUXIN RESPONSE FACTOR condensation and nucleo-cytoplasmic partitioning
Source: Nat Commun. 2022 Jul 11;13:4015. doi: 10.1038/s41467-022-31628-2 (PMC9273615; doi:10.1038/s41467-022-31628-2)
Supplement: Supplementary file 1 — Supplementary Information [file 41467_2022_31628_MOESM1_ESM.pdf]

Supplementary Figures

Regulation of AUXIN RESPONSE FACTOR condensation and nucleo-cytoplasmic partitioning

Hongwei Jing[1,2,3,*], David A. Korasick[4,*], Ryan J. Emenecker[2,3,4], Nicholas Morffy[1,2,3], Edward G. Wilkinson[1,2,3], Samantha K. Powers[4], Lucia C. Strader[1,2,3]

Affiliations:
1. Department of Biology; Duke University; Durham, NC 27008; USA
2. Center for Engineering MechanoBiology; Washington University; St. Louis, MO 63130; USA
3. Center for Science and Engineering Living Systems (CSELS); Washington University; St. Louis, MO 63130; USA
4. Department of Biology; Washington University; St. Louis, MO 63130; USA
* These authors contributed equally to this work

Correspondence: lucia.strader@duke.edu (L.C.S.)

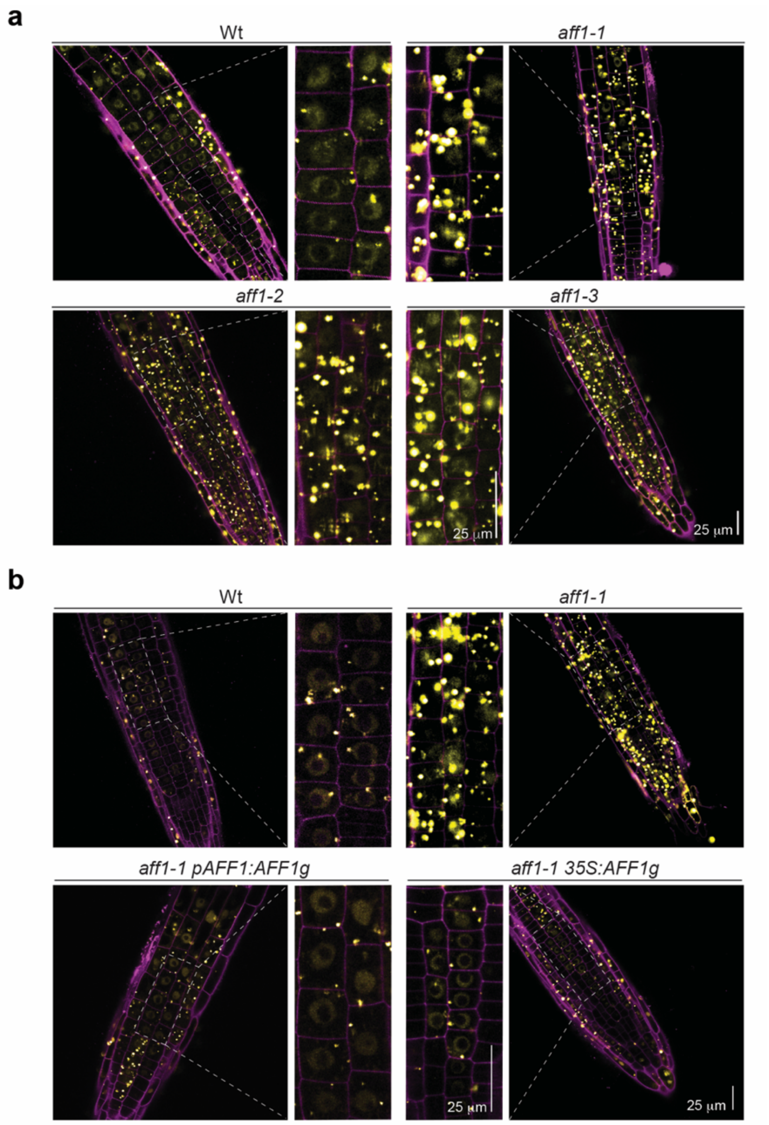

Supplementary Figure 1. AFF1 complement the ARF19 hypercondensation in *aff1-1*. (**a**) Confocal images of 3d-old wild type (Wt; Col-0), *aff1-1*, *aff1-2* and *aff1-3* seedlings carrying *35S:YFP-ARF19* (false-colored yellow) with cell walls counterstained with propidium iodide (false colored magenta). Scale bar = 25 µm. (**b**) Confocal images of 3d-old wild type (Wt; Col-0), *aff1-1*, *aff1-1 pAFF1:AFF1g and aff1-1 35S:AFF1g* seedlings carrying *35S:YFP-ARF19* (false-colored yellow) with cell walls counterstained with propidium iodide (false colored magenta). Scale bar = 25 µm. Three independent experiments were performed for (a) and (b) with similar results.

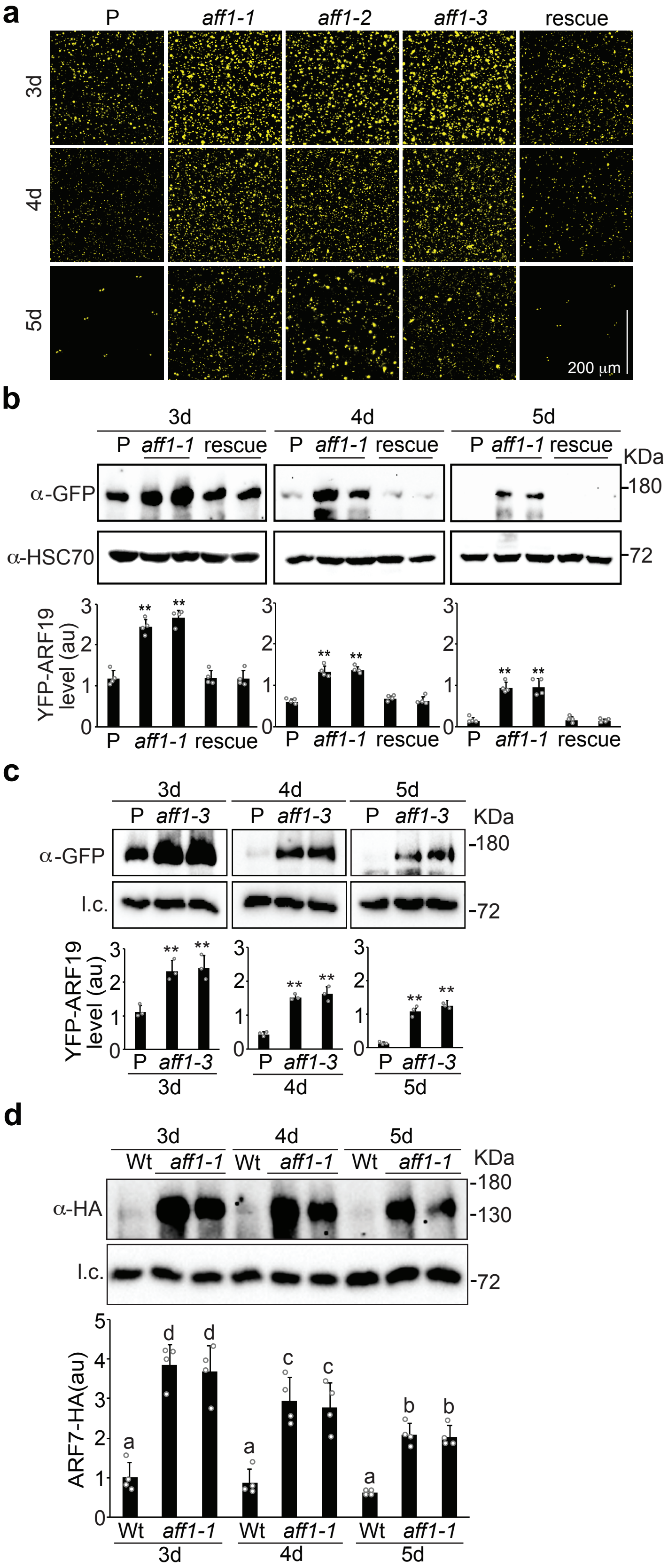

Supplementary Figure 2. AFF1 complement the ARF19 hyperaccumulation in *aff1-1*. (**a**) Confocal microscopy images of 3d-, 4d-, or 5d-old *arf19-1 35S:YFP-ARF19* (P), *aff1-1 35S:YFP-ARF19*, *aff1-2 35S:YFP-ARF19*, and *aff1-3 35S:YFP-ARF19* and rescue line (*aff1-1 arf19-1 35S:YFP-ARF19 pAFF1:AFF1g*) seedling cotyledons. Three independent experiments were performed with similar results. Scale bar = 200 µm. (**b**) Immunoblot analysis of YFP-ARF19 from *arf19-1 35S:YFP-ARF19* (P), *aff1-1 arf19-1 35S:YFP-ARF19*, and two independent rescue lines (*aff1-1 arf19-1 35S:YFP-ARF19 pAFF1:AFF1g*). Quantitative analysis of the relative levels of YFP-ARF19 proteins is presented below the blots. Data are mean ± SD from four independent experiments and gray dots represent the individual values. The statistical significance was determined by a two-sided Student's *t*-test (Paired two sample for means). *P* values = 0.0018 (*aff1-1*_3d), 0.00075 (*aff1-1*_3d), 0.7589 (rescue_3d), 0.8246 (rescue_3d), 0.00089 (*aff1-1*_4d), 0.00107 (*aff1-1*_4d), 0.0965 (rescue_4d), 0.8200 (rescue_4d), 0.00235 (*aff1-1*_5d), and 0.0078 (*aff1-1*_5d), 0.4668 (rescue_5d), 0.8322 (rescue_5d). ** $P < 0.01$ when compared to P. (**c**) Immunoblot analysis (top) and quantification (bottom) of 3d-, 4d-, or 5d-old wild type (Wt; Col-0) or *aff1-3* seedlings carrying *35S:YFP-ARF19*. Anti-GFP antibodies were used to detect YFP-ARF19, and anti-HSC70 antibodies were used to detect HSC70 (l.c.; loading control). Data are mean ± SD from three independent experiments and gray dots represent the individual values. The statistical significance was determined by a two-sided Student's *t*-test (Paired two sample for means). *P* values = 0.0036

(*aff1-3*_3d), 0.0071 (*aff1-3*_3d), 0.0014 (*aff1-3*_4d), 0.0013 (*aff1-3*_4d), 0.0014 (*aff1-3*_5d), and 0.0037 (*aff1-5*_5d). ** $P < 0.01$ when compared to P. (**d**) Immunoblot analysis (top) and quantification (bottom) of 3d-, 4d-, or 5d-old wild type (Wt; Col-0) or *aff1-1* seedlings carrying *35S:ARF7-HA*. Anti- HA antibodies were used to detect ARF7-HA, and anti-HSC70 antibodies were used to detect HSC70 (l.c.; loading control). Data are mean ± SD from four independent experiments and gray dots represent the individual values. Different letters indicate individual groups for multiple comparisons with significant differences (one-way ANOVA, Duncan, $p < 0.05$). The source data in (b), (c) and (d) are provided as a Source Data file.

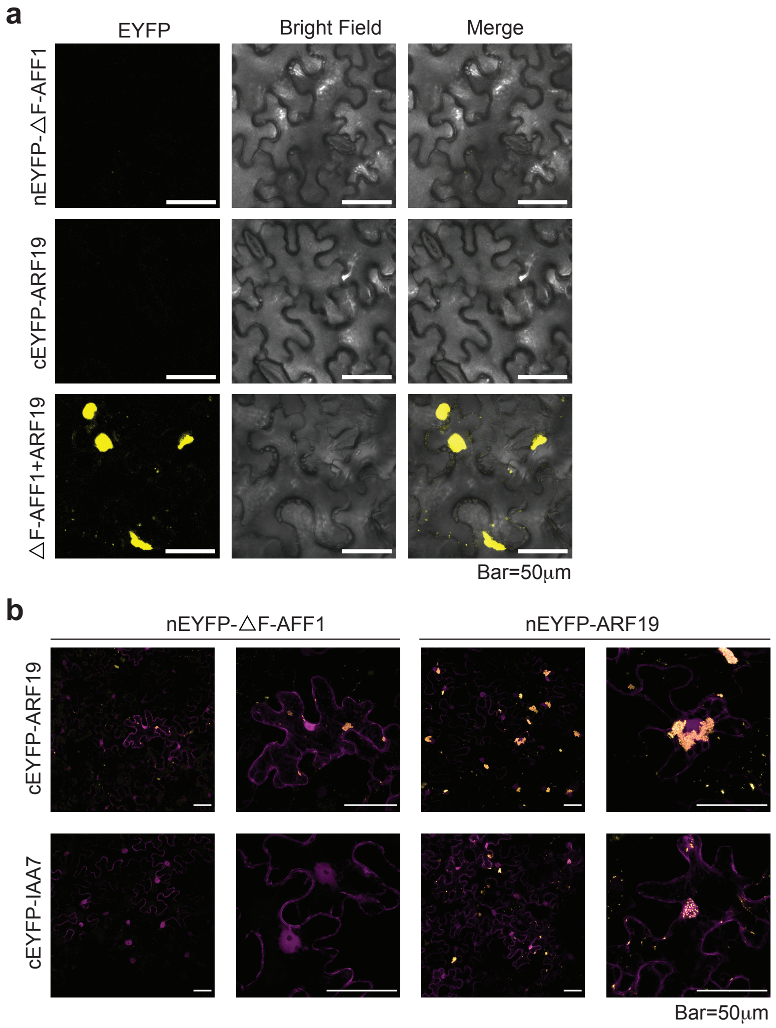

Supplementary Figure 3. Interaction of ARF19 with ΔF-box-AFF1 using BiFC experiment. (**a**) BiFC assays were used to determine the interaction between ARF19 with ΔF-box-AFF1 when transiently expressed in tobacco leaves. nEYFP-ΔF-box-AFF1C denotes expression of the EYFP N-terminal fusion with ΔF-box-AFF1C construct. cEYFP-ARF19C denotes expression of the EYFP C-terminal fusion with ARF19C construct. Scale bar = 50 μm. (**b**) BiFC assays were used to analysis the interaction between nEYFP-ΔF-box-AFF1 and cEYFP-ARF19, between nEYFP-ΔF-box-AFF1 and cEYFP-IAA7, between nEYFP-ARF19 and cEYFP-ARF19, and between nEYFP-ARF19 and cEYFP-IAA7. Nuclear marker WPP-mCherry was co-expressed in the tobacco leaves. Scale bar = 50 μm. Right panel images of each treatment were same as the Figure 5e. Three independent experiments were performed for (a) and (b) with similar results.

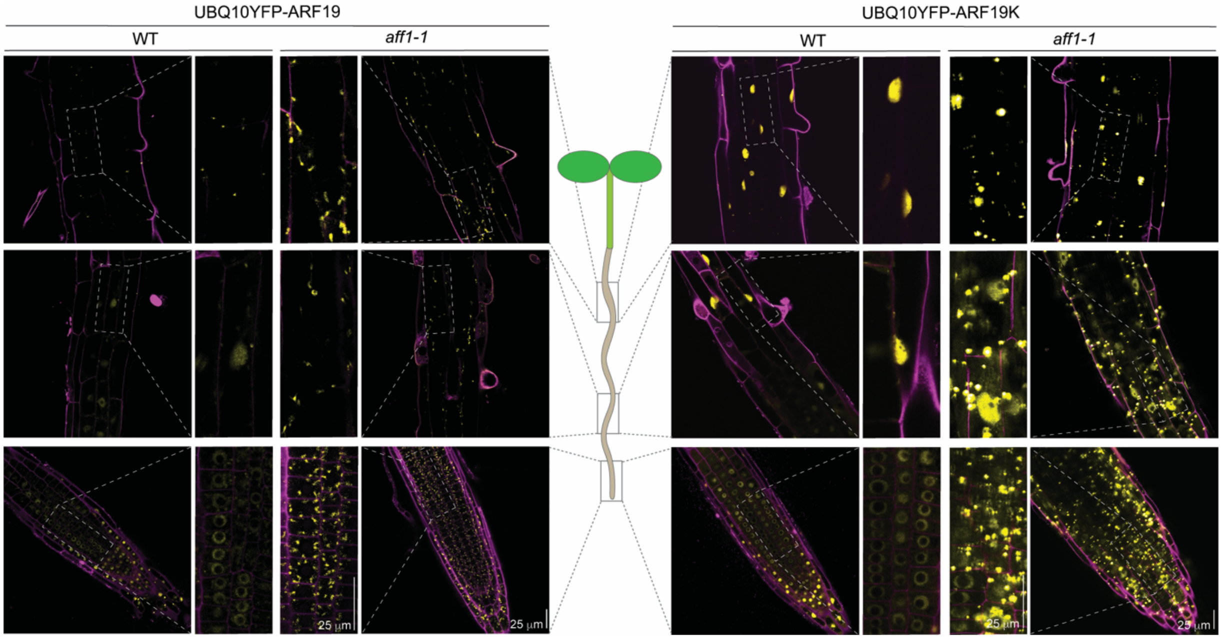

Supplementary Figure 4. ARF19 and ARF19^{K962A} form condensates in all root cells of *aff1-1*. Confocal images of upper root, intermediate and root tip sections from 3d-old wild type (Wt; Col-0) or *aff1-1* seedlings carrying *UBQ10:YFP-ARF19* or *UBQ10:YFP-ARF19^{K962A}* (false-colored yellow) with cell walls counterstained with propidium iodide (false-colored magenta). Scale bar = 25 µm. Three independent experiments were performed with similar results.

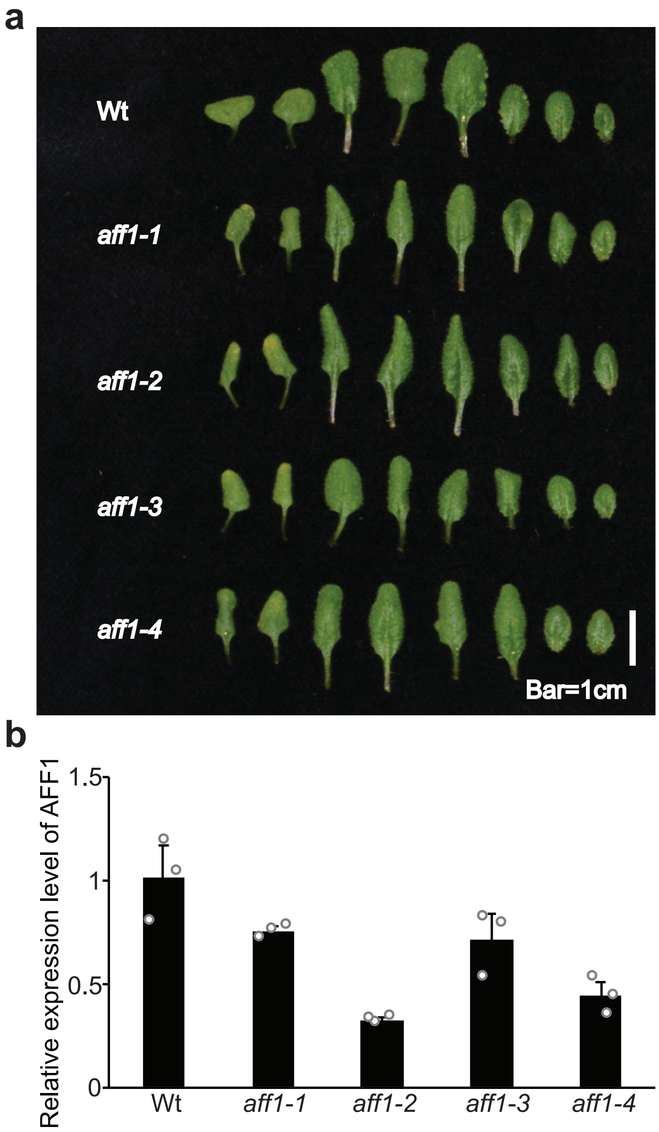

Supplementary Figure 5. *AFF1* mutation exhibits developmental defects. (**a**) Leaf phenotypes of 22d-old wild type (Col-0), *aff1-1*, *aff1-2*, *aff1-3*, and *aff1-4* plants. Scale bar = 1 cm. (**b**) Mean relative accumulation of *AFF1* transcript levels in wild type (Wt; Col-0), *aff1-1*, *aff1-2*, *aff1-3*, and *aff1-4* as assessed by qRT-PCR. Data are mean ± SD from three independent experiments and gray dots represent the individual values. The source data in (b) are provided as a Source Data file.

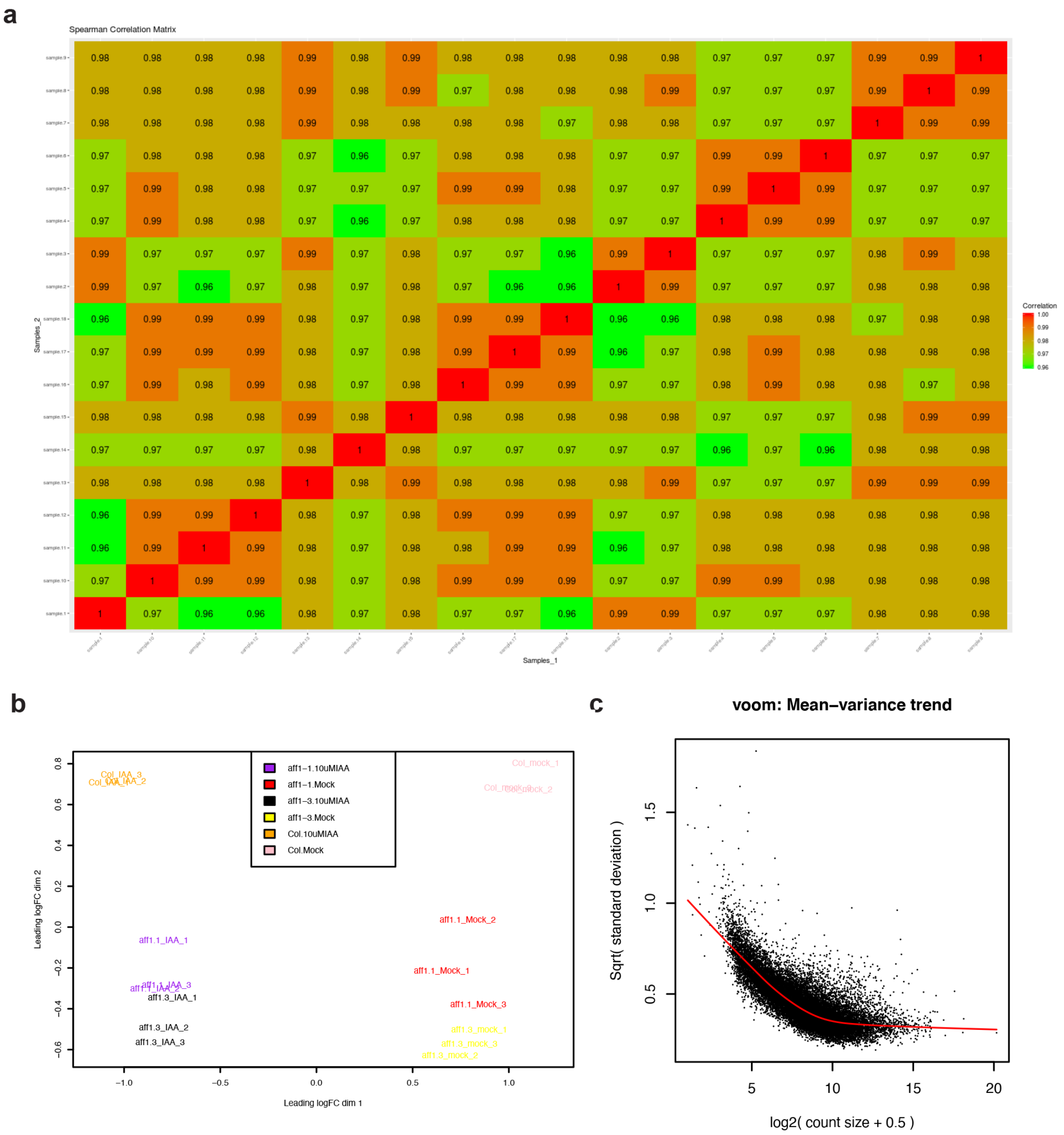

Supplementary Figure 6. RNA-seq sample quality. (**a**) Matrix of Spearman correlations of all detected genes greater than 1 count-per-million in at least 3 samples relative to each other. Samples of the same condition have very high correlations as expected across the diagonal with no outliers. (**b**) The quality of the samples in a multi-dimensional scaling plot of the leading log fold- changes. Samples for each condition cluster very tightly with each other with good cluster separation between samples of different conditions based on expression profiles. (**c**) Scatter plot of the empirically derived fitted and trended mean-variance relationship across all genes.

Supplemental table 1: primers used in this study

Primers	Sequence (5'-3')	Experiments
ARF19_F	CACCCATATGAAAGCTCCATCAAATGG	pENTR-ARF19
ARF19_R	CTCGAGCTATCTGTTGAAAGAAGCTGC	pENTR-ARF19
AFF1-5	CCATAGAAGATTCGGCTTTGAC	*aff1-1* genotyping
AFF1-DdeI	TCTCGGCATGTTAGAGAGAACTTGTTGCTA	*aff1-1* genotyping
AFF1g-F	CACCATGGATATATGTTGCAAAGAT	pENTR-AFF1g
AFF1g-R	AATAGAAAGTAATGCCAATCACAAACAAG	pENTR-AFF1g
AFF1-P1	GAATTCgagctttgaaatcgaacctcatcactaacatcaacgg	pENTR-AFF1p
AFF1-P2	CTCGAGcctttttgaatctactttgacagatctc	pENTR-AFF1p
AFF1-newCDS-ENTR-F	CACCATGGATATATGTTGCAAAGATATAA	pENTR-AFF1CDS
AFF1-newCDS-ENTR-R	TTAGACCTCGGGGATTATATAGACTGT	pENTR-AFF1CDS
AFF1-newCDS-noFbox-ENTR-F	CACCGTAGTATATCTTAATCCTGACG	pENTR-ΔF AFF1CDS
AFF1-newCDS-BamHI-F	CC GGATCC ATGGATATATGTTGCAAAGATATAA	pGEX4T-1-AFF1
AFF1-newCDS-SalI-R	CC GTCGAC TTAGACCTCGGGGATTATATAGACTGT	pGEX4T-1-AFF1
AFF1-newCDS-noFbox-BamHI-F	CC GGATCC GTAGTATATCTTAATCCTGACG	pGEX4T-1-ΔF AFF1
AFF1-2	CTGTCAAAGTAGATTCAAAAAGGATGG	qRT-PCR
AFF1-3	AGTGGCAGCATCTATTTGCTTTC	qRT-PCR
AFF1-4	GATATAATTAGTGATCTACCAGAAG	qRT-PCR
AFF1C-R-264	AAACTCAATACTCTATCAACGAA	qRT-PCR
AFF1C-R-360	GCGTTCCATCACATTTGATATCCA	qRT-PCR
ACT7F2	TCCATGAAACAACTTACAACTCCATCA	qRT-PCR
ACT7B2	CATCGTACTCACTCTTTGAAATCCACA	qRT-PCR
AFF1-newCDS-noTAA-ENTR-R	GACCTCGGGGATTATATAGACTGT	pENTR-AFF1CDS
AFF1-noF-ATG-newCDS-ENTR-F	CACCATGGTAGTATATCTTAATCCTGACG	pENTR-AFF1CDS
ASK1-F-CGC	CGCGGATCCATGTCTGCGAAGAAGATTGTGTTGA	pET28a-ASK1
ASK1-R-CCC	CCCAAGCTTCAGCTGTCATTCAAAAGCCCATTGGTTCT	pET28a-ASK1
Salk_053818C-LP	TTTTTGCTTTTTGCTTTTTGC	*aff1-2* genotyping
Salk_053818C-RP	AAAGCCGTTATCACTGTGTCG	*aff1-2* genotyping
SALK_083453-LP2	TGAGCAAGGCACTGGTTCATC	*aff1-3* genotyping
SALK_083453-RP2	GACTGTGCATTTCGACGAGGTT	*aff1-3* genotyping
SAIL_427_G06-LP	TGGCAGCATCTATTTGCTTTC	*aff1-4* genotyping
SAIL_427_G06-RP	TAGACACCCGTCAACATCCTC	*aff1-4* genotyping
IAA7 F SalI	caccggtcgacggATGATCGGCCAACTTATGAACC	BiFC
IAA7 R NotI	gcggccgcTCAAGATCTGTTCTTGCAGTAC	BiFC
WPP-mCherry F	caccGGTACCacgcgattg	BiFC
WPP-mCherry R	catctagtaacatagatgacaccgc	BiFC
