# Supplementary material for: Regulation of AUXIN RESPONSE FACTOR condensation and nucleo-cytoplasmic partitioning
Source: Nat Commun. 2022 Jul 11;13:4015. doi: 10.1038/s41467-022-31628-2 (PMC9273615; doi:10.1038/s41467-022-31628-2)
Supplement: Supplementary file 2 — Reporting Summary [file 41467_2022_31628_MOESM2_ESM.pdf]

nature portfolio

Corresponding author(s): Lucia C. Strader

Last updated by author(s): Jun 9, 2022

Reporting Summary

Nature Portfolio wishes to improve the reproducibility of the work that we publish. This form provides structure for consistency and transparency in reporting. For further information on Nature Portfolio policies, see our Editorial Policies and the Editorial Policy Checklist.

Statistics

For all statistical analyses, confirm that the following items are present in the figure legend, table legend, main text, or Methods section.

n/a	Confirmed	
☐	☒	The exact sample size (n) for each experimental group/condition, given as a discrete number and unit of measurement
☐	☒	A statement on whether measurements were taken from distinct samples or whether the same sample was measured repeatedly
☐	☒	The statistical test(s) used AND whether they are one- or two-sided<br>*Only common tests should be described solely by name; describe more complex techniques in the Methods section.*
☐	☒	A description of all covariates tested
☐	☒	A description of any assumptions or corrections, such as tests of normality and adjustment for multiple comparisons
☐	☒	A full description of the statistical parameters including central tendency (e.g. means) or other basic estimates (e.g. regression coefficient) AND variation (e.g. standard deviation) or associated estimates of uncertainty (e.g. confidence intervals)
☐	☒	For null hypothesis testing, the test statistic (e.g. F, t, r) with confidence intervals, effect sizes, degrees of freedom and P value noted<br>*Give P values as exact values whenever suitable.*
☒	☐	For Bayesian analysis, information on the choice of priors and Markov chain Monte Carlo settings
☒	☐	For hierarchical and complex designs, identification of the appropriate level for tests and full reporting of outcomes
☒	☐	Estimates of effect sizes (e.g. Cohen's d, Pearson's r), indicating how they were calculated

Our web collection on statistics for biologists contains articles on many of the points above.

Software and code

Policy information about availability of computer code

Data collection	*Provide a description of all commercial, open source and custom code used to collect the data in this study, specifying the version used OR state that no software was used.*
Data analysis	We have provided a detailed description of analysis of our RNASeq data.

For manuscripts utilizing custom algorithms or software that are central to the research but not yet described in published literature, software must be made available to editors and reviewers. We strongly encourage code deposition in a community repository (e.g. GitHub). See the Nature Portfolio guidelines for submitting code & software for further information.

Data

Policy information about availability of data

All manuscripts must include a data availability statement. This statement should provide the following information, where applicable:

- Accession codes, unique identifiers, or web links for publicly available datasets
- A description of any restrictions on data availability
- For clinical datasets or third party data, please ensure that the statement adheres to our policy

The RNA-seq data discussed in this publication have been deposited in NCBI's Gene Expression Omnibus and are accessible through GEO Series accession number GEO: GSE172353.
Source data are provided with this paper.

Field-specific reporting

Please select the one below that is the best fit for your research. If you are not sure, read the appropriate sections before making your selection.

☒ Life sciences ☐ Behavioural & social sciences ☐ Ecological, evolutionary & environmental sciences

For a reference copy of the document with all sections, see nature.com/documents/nr-reporting-summary-flat.pdf

Life sciences study design

All studies must disclose on these points even when the disclosure is negative.

Sample size	Sample sizes were described in legends and materials and methods for each experiment.
Data exclusions	No data were excluded
Replication	Each experiment was performed a minimum of three independent times.
Randomization	No randomization necessary
Blinding	No blinding was performed; however, experiments were performed by independent members of the lab.

Reporting for specific materials, systems and methods

We require information from authors about some types of materials, experimental systems and methods used in many studies. Here, indicate whether each material, system or method listed is relevant to your study. If you are not sure if a list item applies to your research, read the appropriate section before selecting a response.

Materials & experimental systems

n/a	Involved in the study	
☐	☒	Antibodies
☒	☐	Eukaryotic cell lines
☒	☐	Palaeontology and archaeology
☒	☐	Animals and other organisms
☒	☐	Human research participants
☒	☐	Clinical data
☒	☐	Dual use research of concern

Methods

n/a	Involved in the study	
☒	☐	ChIP-seq
☒	☐	Flow cytometry
☒	☐	MRI-based neuroimaging

Antibodies

Antibodies used	Goat anti-mouse IgG-HRP Santa Cruz Cat. #sc-516102<br>Goat anti-rabbit IgG-HRP Jackson Immunochemicals Cat. #111-035-003<br>Mouse anti-GFP-HRP (1E10H7) Fisher Scientific Cat. #50-553-599<br>Anti-GST (B-14) Santa Cruz Cat. #sc-138<br>Mouse anti-HSC70 (plant) (5B7) Enzo Life Sciences Cat. #ADI-SPA-817-D<br>Anti-His (H-15) Santa Cruz Cat. #sc-803<br>Rat anti-HA (3F10) Sigma-Aldrich Cat. #11867423001<br>Donkey anti-Rat Jackson Immunochemicals Cat. #712-035-150<br>Rabbit anti-Histone H3 (nuclear marker) Agrisera Cat.#As10-710
Validation	All antibodies were examined for specificity and cross-reactivity using the appropriate controls (mutants or lines missing the examined transgene).
